# Enhanced growth after extreme wetness compensates for post-drought carbon loss in dry forests

**DOI:** 10.1038/s41467-018-08229-z

**Published:** 2019-01-14

**Authors:** Peng Jiang, Hongyan Liu, Shilong Piao, Philippe Ciais, Xiuchen Wu, Yi Yin, Hongya Wang

**Affiliations:** 1 College of Urban and Environmental Sciences and MOE Laboratory for Earth Surface Processes, Peking University, Beijing, 100871 China; 2 Harvard China Project, School of Engineering and Applied Sciences, Harvard University, Cambridge, MA 02138 USA; 3 IPSL—LSCE, CEA CNRS UVSQ UPSaclay, Centre d’Etudes Orme des Merisiers, 91191 Gif sur Yvette, France; 4 State Key Laboratory of Earth Surface Processes and Resource Ecology, Beijing Normal University, Beijing, 100875 China; 5 Faculty of Geographical Science, Beijing Normal University, Beijing, 100875 China; 6 California Institute of Technology, Pasadena, CA 91125 USA

## Abstract

While many studies have reported that drought events have substantial negative legacy effects on forest growth, it remains unclear whether wetness events conversely have positive growth legacy effects. Here, we report pervasive and substantial growth enhancement after extreme wetness by examining tree radial growth at 1929 forest sites, satellite-derived vegetation greenness, and land surface model simulations. Enhanced growth after extreme wetness lasts for 1 to 5 years and compensates for 93 ± 8% of the growth deficit after extreme drought across global water-limited regions. Remarkable wetness-enhanced growths are observed in dry forests and gymnosperms, whereas the enhanced growths after extreme wetness are much smaller in wet forests and angiosperms. Limited or no enhanced growths are simulated by the land surface models after extreme wetness. These findings provide new evidence for improving climate-vegetation models to include the legacy effects of both drought and wet climate extremes.

## Abstract (web summary)

Increased extreme wet and dry years and forest growth loss from drought legacy effect lead to a question whether wetness events can conversely compensate for this loss. Here the authors report substantial growth enhancement after extreme wetness compensating for drought-induced growth loss globally.

## Introduction

Global warming has intensified the hydrological cycle, causing shifting rainfall patterns (in space and time) with more frequent extreme wet and/or dry years^1–6^. Extreme wet years are predicted to increase both in frequency and intensity^1,7^. Changes in the regime of extreme drought^4,5^ and extreme wetness^1,7,8^ could have profound impacts on forests^9–13^ and their carbon balance^14^ both locally and globally.

Forests cover around one-third of the global land surface, store about half of the terrestrial carbon^15^ and are the dominating contributors of terrestrial net primary production^16^. Forests not only provide ecological, economic, and esthetic services to humankind^17^ but also mitigate climate warming though evaporative cooling^18^ and carbon sequestration^15^. Nonetheless, the fate of forests under climate change with increasing extremes remains uncertain^14^. Anderregg et al.^12^ analyzed the legacy effects of extreme droughts on tree radial growth using tree-ring records and models and inferred that carbon storage could decrease as a result of extreme droughts in semi-arid forests. The counterpart of extreme drought is extreme wetness, but how forests respond to the latter^1,7,8^ has been less studied.

Field studies have investigated the legacy effects of precipitation on ecosystem functioning^19–21^. Based on a synthesis of long-term series of aboveground primary productivity across diverse ecosystem types, it was shown that previous-year precipitation imposed substantial legacy impacts on current-year production in grasslands and shrubs^21^. In some cases, the lagged response to precipitation and temperature anomalies can last for 36–57 months for tree radial growth, and high-antecedent precipitation improves tree radial growth, e.g., in a *Pinus edulis* (pinyon pine) forest in Colorado^20^. Nonetheless, the scarcity and relatively short duration of field experiments make it difficult to obtain a full picture of how forests respond to extreme wetness, a response that likely differs among regions and ecosystems. In this study, we analyze 1929 stand-level tree-ring chronologies, containing 83,107 site-years across the globe covering the period of 1948–2013, complemented by 32-year of global satellite-derived Normalized Difference Vegetation Index (NDVI) data together with land surface model simulations from the Coupled Model Intercomparison Project, Phase 5 (CMIP5), to investigate the growth response of forests after extreme wetness across the global water-limited regions, i.e., tree-ring sites and/or NDVI pixels showing significant (*p* < 0.05) and positive correlation with climate indices (see Methods), over the past six decades.

We investigated tree radial growth after extreme wetness and compared the legacy effects of extreme wetness and droughts. Extreme wetness and droughts were defined as years when climate indices related to water availability exceeded the 95th quantile and were below the 5th quantile of their site-level distribution during the period of coverage of each data set, respectively. Tree-ring width master chronologies were obtained from the International Tree-Ring Data Bank^22^. Only chronologies with > 25 years of data within the period of 1948–2013 were selected. We defined the legacy effects of extreme wetness and drought as the difference between actual (ring width index) and predicted growth (based on a linear climate-growth model, see Methods) after extreme wetness and drought^12,13^. The three climate indices to quantify the extreme droughts and extreme wetness are Standardized Precipitation-Evapotranspiration Index (SPEI)^23^, precipitation minus potential evapotranspiration (P−PET)^24,25^, and soil moisture from a model reanalysis^26^ (for the 0–100 cm soil depth, all averaged over each year, see Methods). To avoid overlapping effects of extreme wetness and drought, only isolated extreme wetness and drought events (one event within a 5-year window) were considered. To extend the analysis of pointwise tree-ring time series, we also investigated NDVI changes after extreme wetness and extreme drought using the same protocol. NDVI is not equal to tree radial growth but is used here as a proxy of productivity^27^. Further, we calculated effects of antecedent extreme wetness on current-year wood production from the output (wood biomass carbon per unit land area, see Methods) of six land surface models from the CMIP5. Specifically, we aimed to answer the following questions: Whether and to what extent tree radial growths change after extreme wetness? If tree stems experience enhanced growth after extreme wetness, can this growth enhancement compensate for the drought legacy effects across the global water-limited regions? Are the observed growth responses to extreme wetness captured by CMIP5 land surface models?

We found that substantial enhanced radial growth after extreme wetness lasted for 1–5 years, and mostly compensated for the growth deficit from drought legacy effect. In detail, the enhanced radial growths were greater in dry forests and gymnosperms. By contrast, no obvious positive response after extreme wetness was captured by CMIP5 models.

## Results

### Substantial radial growth enhancement after extreme wetness

**Fig. 1:**
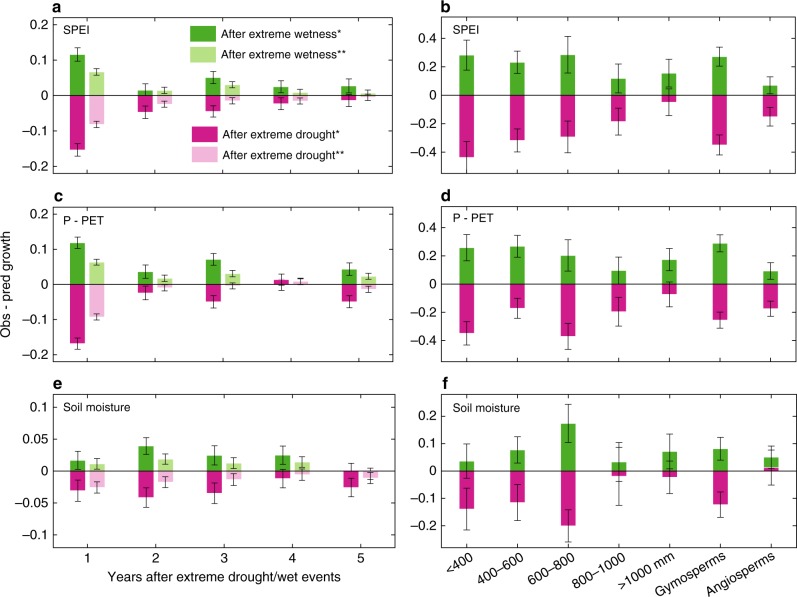
Substantial enhanced radial growth after extreme wetness. Enhanced/reduced growth (unitless) after extreme wet/drought years was observed across 1929 tree-ring chronologies using the SPEI **a**, P−PET **c**, and soil moisture (for the 0–100 cm soil depth) **e**. Enhanced growth across sites that were significantly correlated with climate indices were reclassified into different mean annual precipitation bins and gymnosperm and angiosperm forest types **b**, **d**, **f**. Error bars represent the 95% confidence interval around the mean from bootstrapping (*n* = 5000 resamplings). *: tree-ring sites that were significantly correlated with climate indices (*n* = 631,612,773 for the SPEI, P−PET, and soil moisture, respectively) **: all tree-ring sites (*n* = 1929). P−PET: precipitation minus potential evapotranspiration, SSPEI: Standardized Precipitation-Evapotranspiration Index

We found that enhanced growth lasted 1–5 years after extreme wetness in the tree-ring chronologies (Fig. 1). The magnitude of enhanced tree radial growth to wetness was found to be similar when using the SPEI (22% ± 7% increase in growth above normal conditions, calculated by the difference between observed and predicted growth with a linear model, integrated value of the whole enhanced growth period) and P−PET (21% ± 6%), but a smaller increase (10% ± 5%) was found using soil moisture. The enhanced growth duration differed according to the choice of climate indices, with 5 years for the SPEI, 3–5 years for the P−PET, and 3 years for soil moisture. These results indicate that both the duration and magnitude of enhanced growth after extreme wetness depend upon the water availability indices used to characterize water availability but give broadly consistent (all enhanced growth on average) results. However, the relative magnitude of enhanced growth after extreme wetness was globally (across all sites in different regions) comparable to the magnitudes of reduced growth after extreme drought for the three water availability indices (Supplementary Table 1). The mean compensation ratio (enhanced growth after wetness divided by reduced growth after drought) was 93 ± 8%, suggesting that, on average globally, extreme wetness has offset the negative impacts of extreme drought when considering tree-ring growth anomalies (not account for stand mortality, as drought-induced mortality is generally not sampled or reported by tree-ring records).

Growth changes caused by extreme wetness were also detected using partial autocorrelation analysis (Supplementary Fig. 1). Enhanced growth was consistently observed regardless of the climate indices used (see above and Supplementary Fig. 2, Supplementary Fig. 3) or the percentile thresholds adopted to define extreme wet years (Supplementary Fig. 4). An additional null linear model (i.e., randomly selected the same number years as fake extreme wetness to calculate the growth change pattern, see Methods) indicated that non-climatic drivers (e.g., CO_2_ fertilization, nutrient availability, and disturbance history) of the positive autocorrelation in the chronologies did not significantly influence the inferred enhanced growth (Supplementary Fig. 5). To test whether the increasing nitrogen (N) deposition and/or rising CO_2_ influences our findings, we further analyzed post-wetness and post-drought effects at three epochs (~ 20 years each epoch) in the last 60 years. We found the tree growth recovery from extreme events is getting faster during last six decades, but the enhanced tree radial growth after extreme wetness all sufficiently compensated for the growth deficit after extreme drought at the three epochs (Supplementary Fig. 6), suggesting the trends of N and CO_2_ also do not change our findings. The magnitude of enhanced growth after extreme wet events was negatively correlated with mean background annual precipitation (*r* = −0.13, *p* < 0.05) but not with temperature (*r* = −0.03, *p* = 0.50) nor with the intensity of the extreme wetness (*r* = 0.00, *p* = 0.93) (Supplementary Fig. 7). These results suggest that the enhanced growth after extreme wetness is higher in regions with lower mean annual precipitation.

### Higher enhanced radial growth observed in dry forests

The enhanced growth after extreme wetness in dry forests (with regional mean annual precipitation < 400 mm, regardless of the complex topography-dependent forest distribution) was significantly higher than that in wet forests (mean annual precipitation > 800 mm) based on the SPEI (24% ± 8% vs. 13% ± 6%, *t* = 2.01, *p* < 0.05) and P−PET (29% ± 12% vs. 3% ± 3%, *t* = 4.06, *p* < 0.01) (Fig. 1b, d) and slightly higher when using soil moisture (5% ±5% vs. 4% ± 6%, *t* = 0.5, *p* > 0.1) (Fig. 1f). Reduced growth after extreme drought was also significantly larger in dry forests than that in wet forests (Supplementary Table 2). Enhanced growth anomalies after extreme wetness and reduced growth anomalies after extreme drought in dry forest ecosystems were thus both greater in magnitude than those in wet forests, indicating that trees growing in dry regions are more sensitive to fluctuations in moisture condition (Fig. 1b, d, f). Similar results were generated when using only precipitation instead of water availability indices (Supplementary Fig. 8). Nevertheless, the enhanced growth anomalies after extreme wetness were smaller than the reduced growth anomalies after extreme drought in dry forests for all three climate indices, and the reverse was true in the wet forests (Fig. 1b, d, f).

### Greater enhanced radial growth observed in gymnosperms

The enhanced growth after extreme wetness was significantly greater for gymnosperms than angiosperms (Fig. 1b, d, f). But enhanced growth anomalies after extreme wetness were comparable to reduced growth anomalies after extreme drought in gymnosperms for all three climate indices. In contrast, enhanced growth anomalies after extreme wetness were smaller than the reduced growth anomalies after extreme drought for angiosperms when using SPEI and P−PET, implying adequate compensation for gymnosperms but not for the angiosperms.

The gymnosperms in both wet and dry regions showed substantially greater enhanced growth than that in angiosperms (Supplementary Fig. 9), which probably was associated with their intrinsic hydraulic or nutrient utilization characteristics. By contrast, much smaller enhanced growth after extreme wetness was found for the angiosperms in wet regions, and even slightly reduced growth after extreme wetness was observed for the angiosperms in dry regions (Supplementary Fig. 9). Sufficient compensation was found in the gymnosperms in wet regions (Supplementary Fig. 9). Within the gymnosperms, greater enhanced growth was observed in Cupressaceae than in the other main gymnosperm family, Pinaceae (Supplementary Fig. 10). Meanwhile, the reduced growth in Cupressaceae after extreme drought was smaller than that in Pinaceae, leading to sufficient compensation for Cupressaceae but insufficient compensation for Pinaceae.

**Fig. 2:**
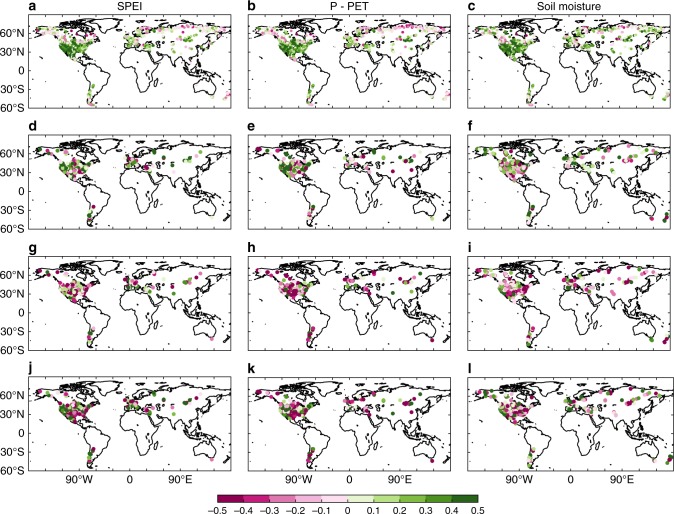
Spatial distribution of tree radial growth change after extreme climate events. Spatial pattern of Pearson’s correlation coefficients between tree-ring width and SPEI **a**, P−PET **b**, and soil moisture **c**. Enhanced growth after extreme wetness, reduced growth after extreme drought, and compensation using the SPEI **d**, **g**, **j**, P−PET **e**, **h**, **k**, and soil moisture **f**, **i**, **l**, summed over the first 5 years after extreme wet/drought years, where the tree-ring chronologies are significantly and positively correlated with the climate indices. Maps were created using Matlab R2015b. P−PET: precipitation minus potential evapotranspiration, SPEI: Standardized Precipitation-Evapotranspiration Index

The spatial patterns of Pearson’s correlation coefficients between tree-ring width and the three different climate indices were similar, with high values observed in arid or semi-arid regions, such as mid-western North America, Inner Asia, and southern Europe (Fig. 2a, b, c). The mean Pearson’s correlation coefficient was higher between ring width index and soil moisture (mean *r* = 0.22) compared with those for SPEI (mean *r* = 0.18) and P−PET (mean *r* = 0.16), suggesting that tree radial growth was more closely related to the soil moisture conditions. Significant enhanced growth after extreme wetness was widely observed in dry forests of the Northern Hemisphere (Fig. 2d, e, f), where tree radial growth was strongly controlled by the soil moisture availability (Fig. 2a, b, c). The most prominent enhanced growth after extreme wetness was observed in western North America and in Western Europe (Fig. 2d, e, f). The enhanced growth for SPEI and P−PET was larger than that for soil moisture, and reduced growth after extreme drought using SPEI and P−PET was lower than that for soil moisture (Fig. 2g, h, i); thus, the compensation for SPEI and P−PET was more sufficient than that for soil moisture (Fig. 2j, k, l). Growth suppression after extreme wetness was detected in central North America, for SPEI and P−PET (Fig. 2d, e), and in southern North America for soil moisture (Fig. 2f). By contrast, the strongest drought legacy effects were observed in the southwestern United States and northern Europe (Fig. 2g, h, i). Notably, the absolute values of enhanced growth after extreme wetness were smaller than those for reduced growth after extreme drought in the southwestern North America for all three climate indices, indicating insufficient compensation in this region (Fig. 2j, k, l).

### Enhanced vegetation greenness after extreme wetness

**Fig. 3:**
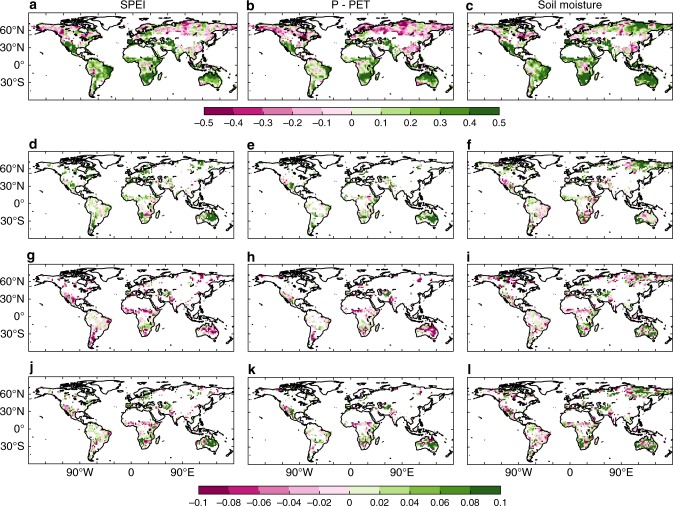
Spatial distribution of NDVI change after extreme climate events. Spatial pattern of Pearson’s correlation coefficients between NDVI_GS_ and the SPEI **a**, P−PET **b**, and soil moisture **c**. The change in greenness after extreme wetness, change in greenness after extreme drought and compensation for the SPEI **d**, **g**, **j**, P−PET **e**, **h**, **k**, and soil moisture **f**, **i**, **l**, where the NDVI_GS_ values are significantly and positively correlated with the climate indices. The pixels with non-woody vegetation, not showing significant and positive correlation with climate indices except for **a**, **b**, **c**, or a multiyear mean NDVI value of < 0.1 were left blank. Maps were created using Matlab R2015b. P−PET: precipitation minus potential evapotranspiration, SPEI: Standardized Precipitation-Evapotranspiration Index

The spatial pattern of Pearson’s correlation coefficients between the NDVI_GS_ (mean NDVI during the growing season, see Methods) and climate indices was similar to that of tree-ring width (Fig. 3a, b, c, Supplementary Fig. 11). Among the studied pixels, 32% pixels were significantly correlated with the soil moisture, 18% were significantly correlated with the P−PET, and 16% were significantly correlated with the SPEI, suggesting that the NDVI_GS_ is also most tightly related to soil moisture. Among the pixels where the NDVI_GS_ was significantly correlated with the climate indices and experienced greenness changes after extreme drought or wetness, the majority of pixels showed enhanced greenness (SPEI, 70%; P−PET, 71%; soil moisture, 58%) after extreme wetness (Fig. 3d, e, f, i). The largest enhancements of greenness after extreme wetness were observed in dry regions (Fig. 3, Supplementary Fig. 12), such as southern African, southwestern North America and Australia, for both the SPEI and P−PET (Fig. 3d, e). Reduced NDVI_GS_ values after extreme drought were also detected in arid and semi-arid regions (Fig. 3g, h). The overall growth compensation of extreme wetness for extreme drought was sufficient for both the SPEI (8% ± 4% vs. −7% ± 3%), P−PET (9% ± 4% vs. −6% ± 3%) and soil moisture (2% ± 2% vs. −1% ± 2%) with prominent compensation effect in Australia, southern South America, and southern Africa (Fig. 3).

### No obvious positive response captured by CMIP5 models

**Fig. 4:**
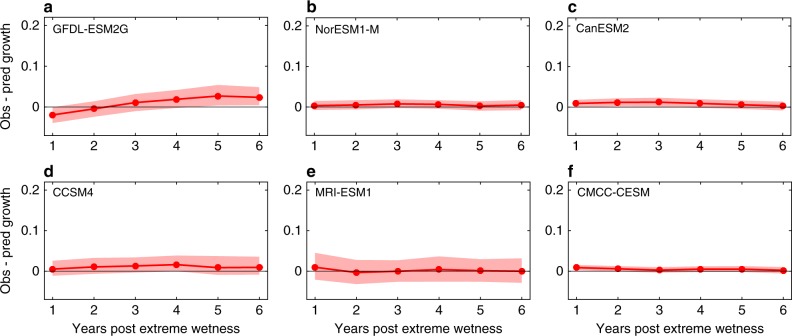
Response to extreme wetness in woody biomass from six CMIP5 models. Growth change patterns after extreme wet years (exceeding the 95th quantile of the annual precipitation forcing data distribution during the analysis period) in the same grid cells in which the tree-ring chronologies were located (those significantly correlated with the climate indices) **a**–**f**. The models used included the Geophysical Fluid Dynamics Laboratory model (GFDL-ESM2G) **a**, the Norwegian Earth System Model (NorESM1-M) **b**, the Canadian Centre for Climate Model (CanESM2) **c**, the Community Climate System Model 4 (CCSM4) **d**, the Meteorological Research Institute model (MRI-ESM1) **e**, and the CMCC-CESM **f**. Shaded regions are the 95% confidence intervals around the mean from bootstrapping (*n* = 5000 resamplings)

Limited or no enhanced growth after extreme wetness years (exceeding the 95th quantile of annual forcing precipitation in each pixel from each model) was found from the CMIP5 model outputs (Fig. 4). The correlation coefficients (mean *r* = 0.05) between wood carbon content (a variable of the CMIP5 output, see Methods) and the climate indices were much smaller than those from tree-ring width. Some models (CanESM2, MRI-ESM1, CMCC-CESM, CCSM4) showed slightly enhanced growth after extreme wetness for 1–5 years (Fig. 4), but this enhanced growth anomaly was very small (mean value < 1%) relative to that diagnosed from tree-ring width and NDVI series. None of the six models from CMIP5 captured the enhanced growth after extreme wetness (Fig. 4), indicating the possibility of lacking some mechanisms to represent the legacy effects of extreme wetness in the current state-of-the-art land surface models.

## Discussion

Our analyses revealed substantially enhanced tree radial growth and enhanced vegetation greenness after extreme wetness years across the global water-limited regions (where tree chronologies and/or NDVI pixels showing significantly positive correlation with climate indices). Previous studies have reported that extreme wetness has a significant impact on forest ecosystems^28^ and that antecedent-year precipitation has substantial legacy effects on whole ecosystem-level carbon fluxes^29^. Moreover, models including antecedent moisture conditions explained an additional 18–28% of the response variation compared to those not considering antecedent effects^20^. However, these studies are limited to few sites or specific ecosystems. Here, we expanded the analysis to much larger temporal and broader spatial scales to gain more knowledge of how different types (dry vs. wet, gymnosperm vs. angiosperm) of forests respond to antecedent extreme wetness.

The effects of extreme wetness have strong and pervasive positive impacts on tree radial growth in the following several years, especially in dry forests. This means that trees, especially in dry environments, are particularly sensitive to extreme wetness pulses and these pulses can largely stimulate tree radial growth. The enhanced tree radial growth after extreme wetness in dry forests has a potentially significant impact on long-term carbon storage and the inter-annual variability of the terrestrial ecosystem carbon cycle. A previous study estimated that the drought legacy effect could lead to a substantial loss of carbon in semi-arid forests^12^, but our study suggests that this may be largely compensated over time by the increased growth after wet years. However, insufficient compensation, that is, negative asymmetry between dry and wet year responses, was detected in some regions (such as southwestern North America) and species (such as angiosperms).

The enhanced tree radial growth after extreme wetness can at least partly be explained by structural and/or biogeochemical carryover effects from the antecedent year. Forest ecosystems tend to have more leaves and roots than average owing to excess carbon storage after extreme wetness^21^, thus allowing more photosynthesis and the exhaustive exploitation of resources (e.g., water, solar radiation, and nutrients) in the following years, leading to enhanced growth. Another possible reason accounting for the enhanced growth is increased nutrient availability, such as increased nitrogen (N) availability, owing to more decomposition by microorganisms in the years following extreme wetness in areas where N availability depends on litter input^21^, facilitating tree radial growth. In addition, as mortality is not recorded or measured by tree-ring records, the potential positive legacy effects (self-thinning effects) for surrounding trees from drought-induced mortality are not assessed in this study. Interestingly, the intuitive explanation for the enhanced growth resulting from soil water carryover from the previous wet year is not the major contributor to the enhanced growth because no significant partial autocorrelation coefficient was found in this study (partial autocorrelation coefficient (PAC) *r* < 0.15, *p* > 0.1), which has also been confirmed by other field studies^19,30,31^. Moreover, the waterlogging of soils damaging anaerobic conditions^31^, and a higher susceptibility of trees to pathogens^21^ reducing tree growth, caused by excessive precipitation are unlikely to occur in dry regions, as the moisture conditions typically are far from sufficient, even in the years after extreme wetness.

In summary, our analyses using multiple climate indices and different vegetation growth indices (tree-ring width and NDVI) consistently showed that forests experienced enhanced growth after extreme wetness, roughly compensating for carbon sink reductions owing to drought legacy effects across the global water-limited regions. This enhanced growth pattern was not captured by the current CMIP5 models, suggesting the weak ability of current land surface models to represent the lagged effects of extreme wetness on the carbon cycle, although increasingly observational studies have reported that previous water conditions have substantial legacy impacts on subsequent vegetation dynamics at local, regional, and global scales^12,19–21,29^. The findings of this study indicate that extreme wetness events have significant and positive hysteresis impacts on forest ecosystem processes. The enhanced growth can at least be partly explained by structural and/or biogeochemical carryover effects, yet studies based on manipulative experiments are needed in the future to clarify the underlying physiological mechanisms.

## Methods

### Tree-ring width chronologies

In total, 1929 standard tree-ring width chronologies were obtained from the International Tree-Ring Data Bank (ITRDB) https://www.ncdc.noaa.gov/data-access/paleoclimatology-data/datasets/tree-ring) on 1 November 2016. The ITRDB is the world’s largest public archive of tree-ring data and is managed by the National Center for Environmental Information (NCEI) Paleoclimatology Team and the World Data Center for Paleoclimatology. Hundreds of research groups and dendrochronologists from all over the world contributed generously to this data bank. The tree-ring samples were collected in the field by using increment borer to get a cylinder of wood along the radius of a tree by investigators. Typically, the minimum threshold is 20 trees per site to get reliable statistical analysis, but this will vary according to the specific characteristics (such as the strength of the climate signal) of the sites and the purpose of the collection. Two samples were generally collected per tree to facilitate cross-correlation and accurate dating of the annual ring. The samples are mounted and finely sanded to allow cross-dating and measured the widths of the annual rings. The ring widths are measured to the ~ 0.01 mm or 0.001 mm and recorded in computerized data files. Then, a statistical evaluation of the cross-dating has been conducted by using the COFECHA program^32^. After that, cross-dated raw ring width measurements are converted to site-level chronologies of standardized ring width indices via detrending (commonly based on modified negative exponential curve fitting or cubic smoothing spline^33^) to remove low-frequency ring width fluctuations related to increasing tree size/age or to stand dynamics by using ARSTAN^34^. There are several types of standardization methods, and we collected the site-level standard chronologies generated by each research group and assumed that those researchers selected the most suitable standardization for their data^12^. We chose these chronologies based on three criteria: they covered at least 25 years during 1948–2013, the basic information (e.g., species name, longitude, latitude, elevation) was complete, and the chronologies had detrended-only files to remove longer-term signals embedded within the raw ring width.

The species distribution and the spatial coverage of those selected chronologies are presented in Supplementary Table 3 and Supplementary Fig. 13, respectively. We found that the forest sites that significantly and positively correlated with water availability indices mainly distributed (61–69% of tree-ring sites, Fig. 2, Supplementary Fig. 13, Supplementary Fig. 14) in drylands^35^ (Aridity index, AI = P/PET, AI < 0.65, Supplementary Fig. 14). Some sites/pixels (15–17% for tree-ring sites) with significant and positive correlation with water availability indices were also found in humid^35^ regions (AI > 1, Fig. 2, Supplementary Fig. 13, Supplementary Fig. 14), which is probably attributable to the specific site conditions, for example, poor water holding capacity of soil property in karst region, and seasonal drought may also have a role in this phenomenon.

### Climate indices

We used three different climate indices to examine the pattern in growth change after extreme wetness, including the Precipitation-Evapotranspiration SPEI^23^, P−PET^24,25^, and soil moisture for the 0–100 cm soil depth^26^.

SPEI data with a spatial resolution of 0.5°, which were calculated based on monthly precipitation and potential evapotranspiration estimated by the Penman-Monteith equation of CRU TS 3.23^36^, were obtained from SPEIbase V2.4 (http://sac.csic.es/spei/database.html). First, the difference between P and PET was calculated for the month *i* using equation 1,

1 $${\mathrm{D}}_{\mathit{i}} = {\mathrm{P}}_{\mathit{i}} - {\mathrm{PET}}_{\mathit{i}}$$

where D_*i*_ is a measure of water surplus or deficit. The D_i_ values were then accumulated to different timescales^36^. The difference ${\mathrm{D}}_{{\mathit{i}},{\mathit{j}}}^{\mathit{k}}$ in a given month *j* and year *i* depends upon the timescale *k*. For example, the aggregated difference for month *j* in year *i* with a 12-month time-scale is calculated using equations 2–3,

2 $${\mathrm{X}}_{{\mathit{i}},{\mathit{j}}}^{\mathit{k}} = \mathop {\sum }\limits_{{\mathit{l}} = 13 - {\mathit{k}} + {\mathit{j}}}^{12} {\mathrm{D}}_{{\mathit{i}} - 1,{\mathit{l}}} + \mathop {\sum }\limits_{{\mathit{l}} = 1}^{\mathit{j}} {\mathrm{D}}_{{\mathit{i}},{\mathit{l}}},{\mathrm{if}}\,j \,< \,k$$

and

3 $${\mathrm{X}}_{{\mathit{i}},{\mathit{j}}}^{\mathit{k}} = \mathop {\sum }\limits_{{\mathit{l}} = {\mathit{j}} - {\mathit{k}} + 1}^{\mathit{j}} {\mathrm{D}}_{{\mathit{i}},{\mathit{l}}},{\mathrm{if}}\,{\mathit{j}} \ge {\mathit{k}}$$

where D_*i*_,_1_ is the difference between P and PET in the first month of year *i*. Finally, the water balance was normalized into a log-logistic probability distribution to obtain the SPEI index series^36^. The parameter estimation^37,38^ of the log-logistic probability distribution and detailed calculation procedure for SPEI index can be found in Vicente-Serrano et al.^36^.

The timescale of SPEI spans from 1 to 48 months in the SPEIbase V2.4. As a multiscale drought and wetness indicator, SPEI was widely used in studying the impact of climate extremes on terrestrial ecosystems^13,39^. Considering that the majority of vegetation types respond predominantly to mean annual SPEI within timescales of 2–4 months^40^ and that the SPEI03 (3-month timescale) was widely used to study the relationship between vegetation dynamics and moisture variability^13^, we chose annual mean SPEI03 as one drought/wetness indicator.

Monthly precipitation and PET data with a spatial resolution of 0.5° were obtained from the National Oceanographic and Atmospheric Administration Precipitation Reconstruction Over Land (NOAA PREC/L)^24^ and CRU TS 3.22^25^, respectively, to calculate the index of P−PET. The precipitation data from PREC/L^24^ rather than CRU TS 3.22 was used to calculate P−PET to gain a different index from SPEI (calculated based on precipitation and PET both from CRU). The PREC/L precipitation data sets (covering the period of 1948–2010) were constructed using the optimal interpolation technique applied to gauge observations from over 17,000 stations collected in the Global Historical Climatology Network and the Climate Anomaly Monitoring System data sets^24^, which can be accessed from https://www.esrl.noaa.gov/psd/data/gridded/data.precl.html. The PET data set were calculated based on the Penman-Monteith equation and were downloaded from the CRU (http://www.cru.uea.ac.uk/). In addition, we obtained the other two precipitation data sets from CRU TS 3.22 and Global Precipitation-Climatology Centre (GPCC^39^, https://climatedataguide.ucar.edu/climate-data/gpcc-global-precipitation-climatology-centre). All the three kinds of precipitation data sets are interpolated based on global precipitation station data.

We used the soil moisture data from model simulation of the Noah Land Surface Model^41^ 3.3 forced by the global meteorological forcing data set from Princeton University^42^ (https://disc.gsfc.nasa.gov/datasets/). The simulation used the common GLDAS data sets for land cover (MCD12Q1)^43^, land water mask (MOD44W)^44^, soil texture^45^, and elevation (GTOPO30). The latest version of simulated soil moisture (GLDAS-2.1 Noah 0.25° products) was used in this study. The spatial resolution of the monthly soil moisture simulation is 0.25°, and we aggregated it to 0.5° to match the other two climate indices. We also calculated soil moisture for the 0–100 cm soil depth based on the original data.

In summary, the three wetness and drought indicators SPEI, P−PET, and soil moisture were obtained/calculated from different data sets; thus, they can be viewed as different and can be compared to evaluate the impacts of climate extremes on forest ecosystems.

To make this study more comprehensive, we also analyzed the effects of extreme events from other commonly used climate indices, including the self-calibration Palmer Drought Severity Index^46^ (scPDSI, http://www.cgd.ucar.edu/cas/catalog/climind/pdsi.html), and the land surface model-simulated soil moisture of the Climate Prediction Center^47^ (CPC, http://www.cpc.ncep.noaa.gov/products/Soilmst_Monitoring/US/Soilmst/Soilmst.shtml). Results of these analyses are presented in supplementary materials (Supplementary Figs. 2–4). The comparison shows that the correlation coefficients between tree radial growth and these different climate indices exhibit similar spatial patterns (Supplementary Fig. 2), with higher correlation coefficients in arid and semi-arid ecosystems. The correlation coefficients between tree radial growth and SPEI03 (with a timescale of 3 months), scPDSI, and CPC soil moisture were relatively higher than those of the three monthly global precipitation data sets (Supplementary Fig. 2). Moreover, a similar enhanced growth pattern after extreme wetness was observed for the different climate indices (Supplementary Fig. 3), strengthening the robustness of our findings.

### Satellite NDVI measurements

The latest version of the biweekly NDVI data set, which was derived from Advanced Very High Resolution Radiometer observations, during 1982 to 2013 were obtained from the Global Inventory Modeling and Mapping Studies (GIMMS, https://climatedataguide.ucar.edu/climate-data/ndvi-normalized-difference-vegetation-index-3rd-generation-nasagfsc-gimms) group (i.e., GIMMS NDVI3g). This data set has been processed to address various deleterious effects, including calibration loss, orbital drift, sensor degradation, intersensor differences, cloud cover, zenith angle, and volcanic aerosols^48^. The GIMMS NDVI3g data set has a spatial resolution of 0.083° (~ 8 km) and was aggregated to a spatial resolution of 0.5° to match the climate indices. It has been widely used to characterize land cover and to monitor spatiotemporal changes in vegetation activity and productivity in response to climate variations and extreme events both regionally and globally^13,27,49–52^. We adopted a simple maximum value compositing (MVC) technique^53^ (obtaining the larger 15-day MVC NDVI for each month to produce monthly NDVI data sets) to minimize atmospheric effects and cloud contamination effects. We calculated the annual mean growing season NDVI for the period of 1982–2013. Following previous studies^13,51^, we roughly defined growing season as April to October for the extratropical Northern Hemisphere (23°N–90°N), October to April for the extratropical Southern Hemisphere (23°S–90°S) and all year for tropical region (23°S–23°N). Pixels with multiyear mean annual NDVI values below 0.1 during 1982 to 2013 were removed from further analyses.

The growth change patterns of different ecosystems after climate extremes differ, and a prolonged response time for deep-rooted forests was detected^13^ using the NDVI3g (~ 4 years for forests, 2 years for shrubs). In this study, we adopted the same protocol used by Wu et al.^13^ to investigate greenness changes from the NDVI3g. We grouped evergreen broadleaf forest, evergreen needle-leaf forest, deciduous needle-leaf forest, deciduous broadleaf forest, and mixed forest as forests; closed shrub lands, open shrub lands, woody savannas, and savannas were categorized as shrubs. As many tree-ring sites were located in shrub areas (Fig. 2, Supplementary Fig. 15), we also investigated the vegetation greenness changes of shrubs (defined in this study) after extreme wetness/drought. In addition, the woody characteristics of the shrubs also facilitated comparison with the woody biomass outputs from the CMIP5. The classification was based on the Moderate Resolution Imaging Spectroradiometer land cover product MOD12C1 (http://glcf.umd.edu/data/lc/). It identifies 17 land cover classes (nine types were used in this study) defined by the International Geosphere-Biosphere Program scheme (Supplementary Fig. 15).

### Woody biomass in the CMIP5 models

Following a previous study^12^, we obtained the wood carbon content per unit land area, precipitation, and soil moisture from historical runs from six land surface model outputs in the CMIP5, https://esgf-node.llnl.gov/projects/cmip5/), multi-model ensemble archive: GFDL-ESM2G, NorESM1-M, CanESM2, CCSM4, MRI-ESM1, and CMCC-CESM. Only one realization of each model is needed because enhanced growth should be insensitive to the initial conditions and be presented in all realizations^12^. We only used the pixels in the model output in which the tree-ring width index was significantly and positively correlated with the climate indices and calculated the growth change pattern after extreme wetness using the same approach as the calculation of the tree radial growth change pattern.

### Growth change pattern

Two methods were used to quantify changes in the tree-ring width chronologies after extreme wetness/drought: the residual value of the observed tree-ring width minus the predicted growth after extreme wetness based on a linear climate-growth relationship and partial autocorrelation analysis. These two effective methods have been used in previous studies to quantify the legacy effects of drought on forest^12^, shrubs, and grass growth^13^.

In the first method, we defined growth change as the difference between the observed tree radial growth and the predicted growth, which was estimated using a linear regression model over the entirely overlapped period between the chronologies and different climate indices:

4 $${\mathrm{G}}_{\mathit{C}} = {\mathrm{G}}_{\mathit{O}} - {\mathrm{G}}_{\mathit{P}}$$

Where G_*c*_ stands for the growth change pattern, G_*O*_ represents the observed tree radial growth, and G_*p*_ stands for the predicted growth estimated by the null linear model.

The second method relied on the calculation of a PAC. The partial autocorrelation function gives the partial correlation of a time series with its own lagged values while conditioning the values of the time series for all shorter times. For example, the partial autocorrelation of order 3 measures the effect (linear dependence) of y_*t*_ on y_*t*+3_ after removing the effect of y_*t*+1_ and y_*t*+2_ on y_*t*+3_:

5 $${\mathrm{y}}_{{\mathit{t}} + 3} = \alpha_0{\mathrm{y}}_{\mathit{t}} + \alpha_1{\mathrm{y}}_{{\mathit{t}} + 1} + \alpha_2{\mathrm{y}}_{{\mathit{t}} + 2} + {\mathrm{\varepsilon }}$$

The estimate of *α*_0_ will give the value of the partial autocorrelation of order 3. Extending the regression with *k* additional lags, the estimate of the last term will give the partial autocorrelation of order *k*.

The null model for predicting growth depends on the strength of the linear regressions between tree radial growth and the climate indices. Given that an uninformative null model (the regression slope does not significantly differ from zero) would give a perfect recovery after climate extremes, we confined our analysis to the sites/grids that showed significant and positive correlations between the climate indices.

To test whether non-climatic drivers (e.g., CO_2_ fertilization, nutrient availability changes, or disturbance history) with potentially positive autocorrelation in chronologies influence the findings of this study, we constructed a random null model. In this random null model, we firstly randomly picked the same number of years for each site than extreme wet years to obtain fake wet extremes. Then, we conducted the same analysis of growth change anomalies after each selected year and repeated this process 5000 times, and concluded to no significant growth change. This suggests that random non-climate effects do not affect our main conclusions (Supplementary Fig. 5). This method was successfully used in previous studies to test random non-climate effects on tree growth^12^.

## Supplementary information

Supplementary Information

## Data Availability

The authors declare that the source data supporting the findings of this study are provided within the paper.
