# Supplementary material for: Enhanced growth after extreme wetness compensates for post-drought carbon loss in dry forests
Source: Nat Commun. 2019 Jan 14;10:195. doi: 10.1038/s41467-018-08229-z (PMC6331561; doi:10.1038/s41467-018-08229-z)
Supplement: Supplementary file 1 — Supplementary Information [file 41467_2018_8229_MOESM1_ESM.pdf]

Supplementary Information for

Enhanced growth after extreme wetness compensates for post-drought carbon loss in dry forests

Jiang et al.

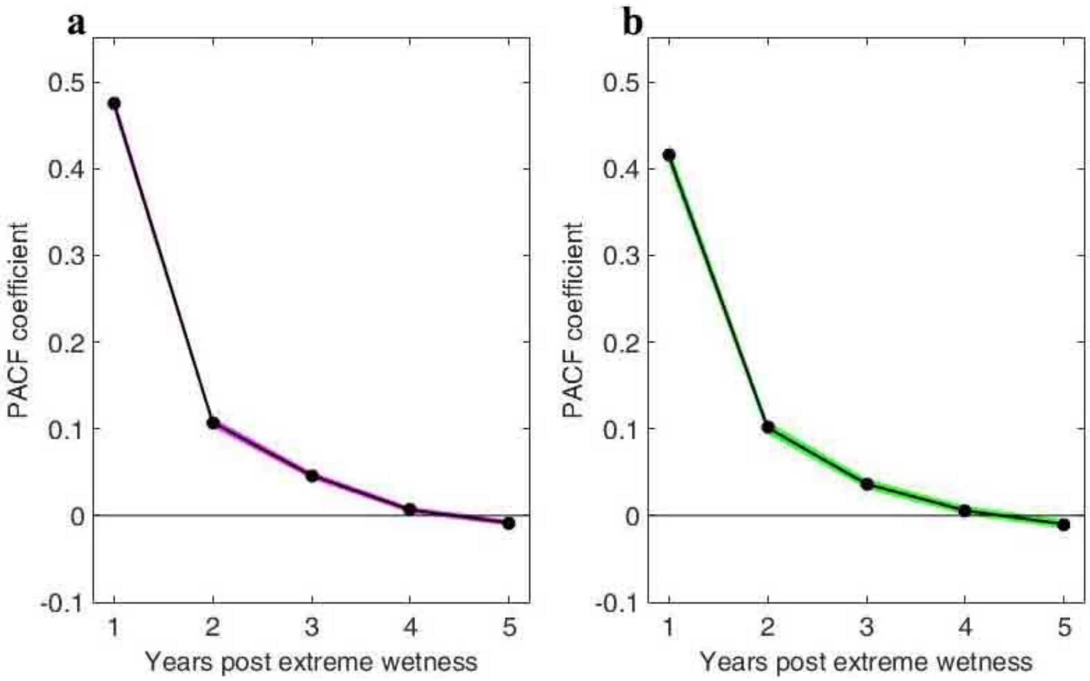

Supplementary Figure 1. Radial growth change in partial autocorrelation function coefficients. Growth change was also detected by PACF. All 1929 chronologies (a) and the chronologies that were significantly correlated (b) with SPEI03 (n = 631). Polygons represent the 95% confidence intervals around the mean from bootstrapping (n = 5000 resamplings).

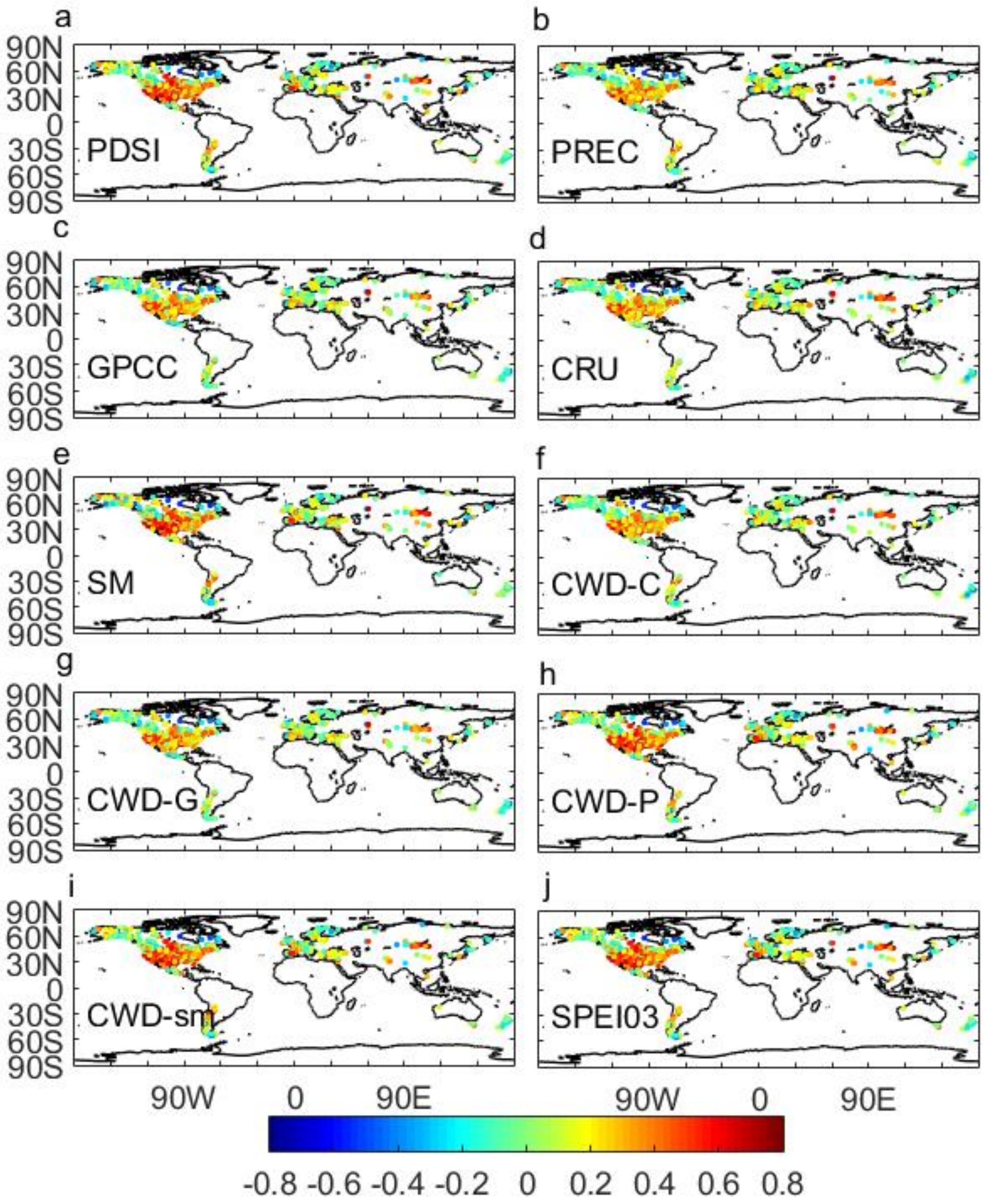

Supplementary Figure 2. Correlation coefficients between different climate indices and radial growth. Different climate indices, PDSI (a), precipitation from PREC (b), GPCC (c), CRU (d), soil moisture (e); CWD-C (f), CWD-G(g), CWD-P (h) and CWD-sm(i), are the differences between the CRU, GPCC, PREC precipitation and soil moisture (CPC) minus the potential evapotranspiration (PET), respectively; SPEI03 (j). Maps were created using Matlab R2015b

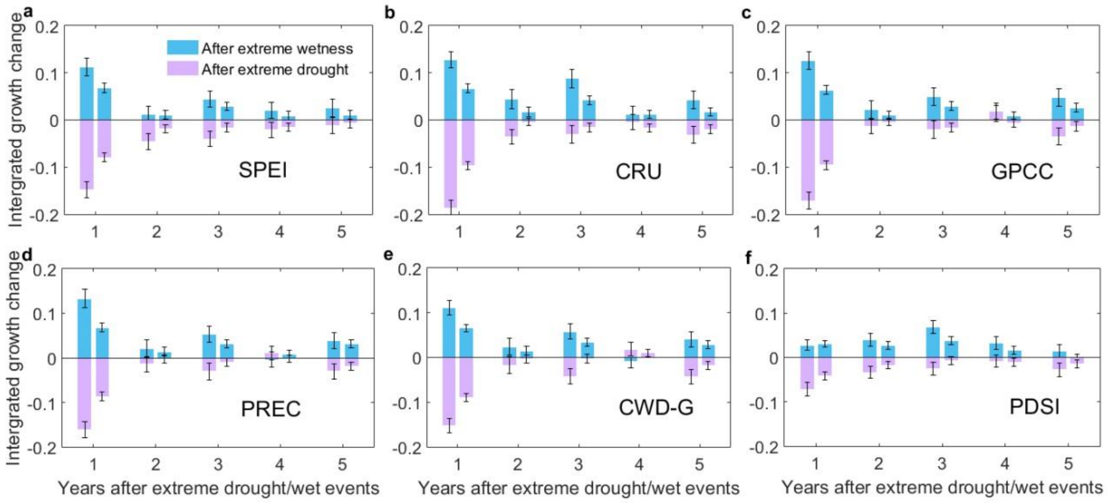

Supplementary Figure 3. Radial growth change using different climate indices. Similar radial growth change pattern was detected regardless of using different climate indices. SPEI (a), precipitation from CRU (a), GPCC (b), PREC (c), soil moisture minus precipitation from GPCC (d), PDSI (e). Error bars represent the 95% confidence intervals around the mean from bootstrapping (n = 5000 resamplings).

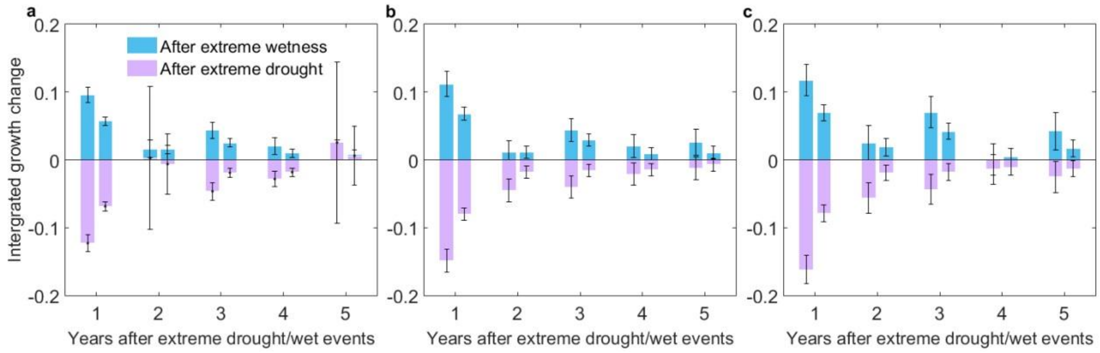

Supplementary Figure 4. Radial growth change using different thresholds. Similar radial growth change pattern was detected regardless of using different thresholds to define extreme climate events.Top and bottom 10th quantile (a), 5th quantile (b), and 1th quantile (c) of the climate index (here SPEI03). Top and bottom represent extreme wetness and extreme drought events, respectively. Error bars represent the 95% confidence intervals around the mean from bootstrapping (n = 5000 resamplings).

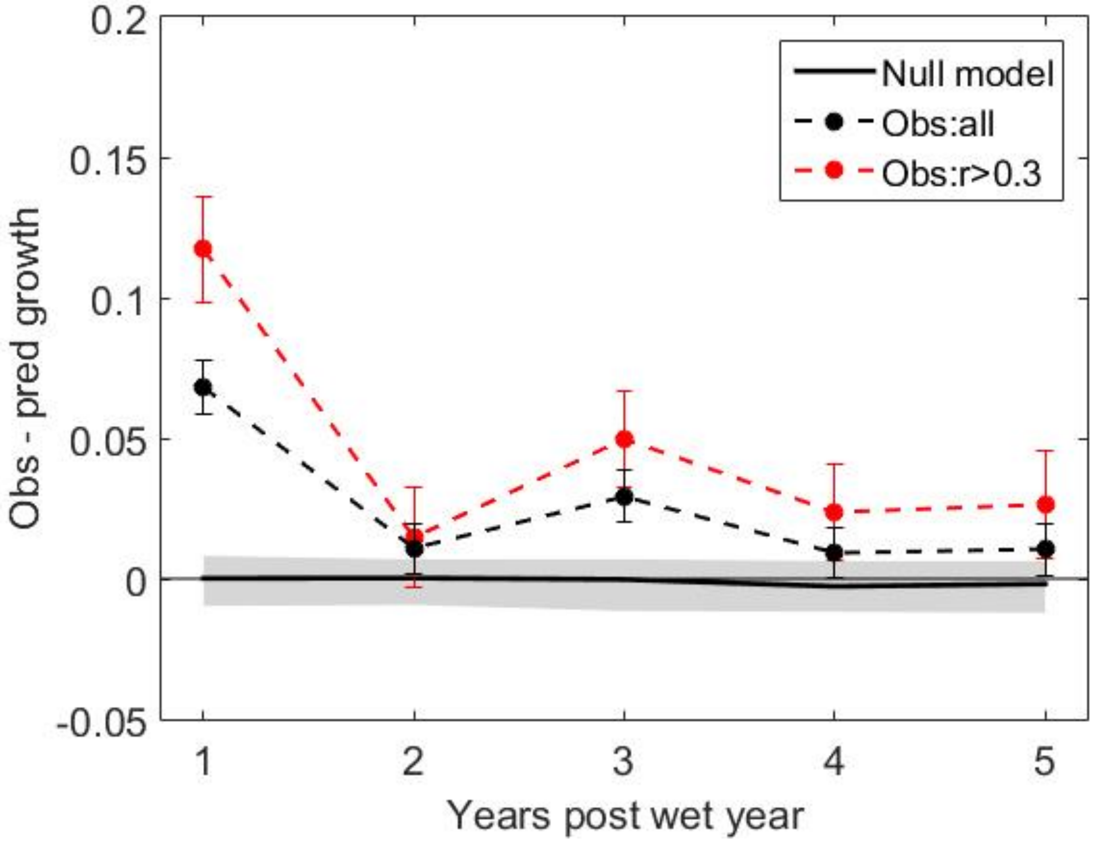

Supplementary Figure 5. Radial growth change in random null model. The radial growth change from random null model was compared to observed change in growth after extreme wetness (exceeding the 95th quantile for SPEI03 value during the analysis period). Error bars and shaded regions represent the 95% confidence intervals around the mean from bootstrapping (n = 5000 resamplings). Black dashed line represents all 1,929 sites, and red dashed line represents the 631 sites that were significantly and positively correlated with the climate indices.

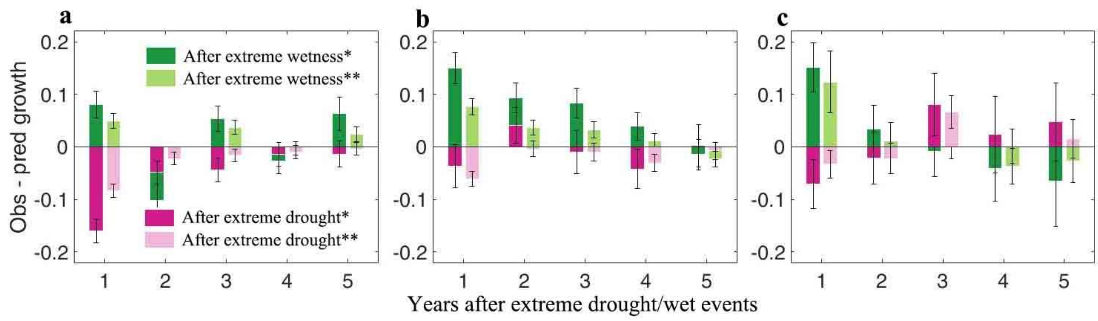

Supplementary Figure 6. Radial growth change at three epochs. Enhanced growth by extreme wetness compensated for growth deficit after extreme drought at all three epochs during the last six decades, 1948-1970 (a), 1970-1990 (b), 1990-2013(c). Error bars represent the 95% confidence intervals around the mean from bootstrapping (n = 5000 resamplings).

*: tree-ring sites that were significantly correlated with climate indices (n = 631, SPEI03)

**: all tree-ring sites (n = 1929)

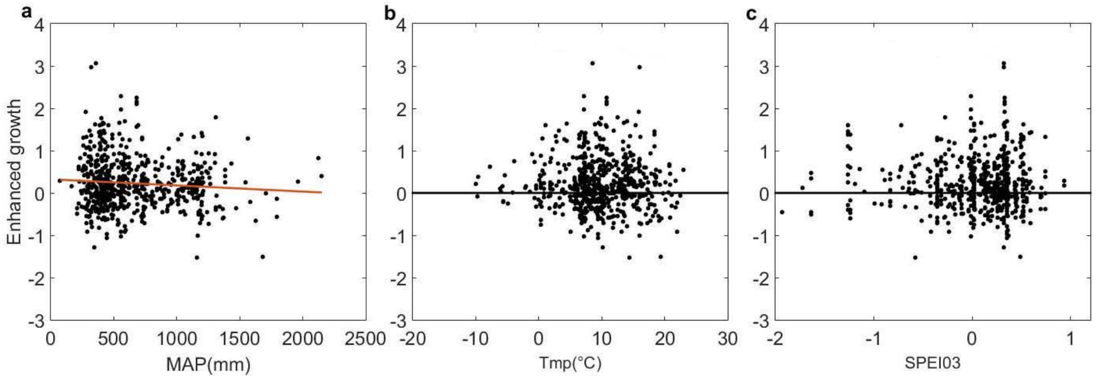

Supplementary Figure 7. Radial growth change correlated with climate. Radial growth change after extreme wetness are negatively associated with mean annual precipitation. The magnitude of enhanced growth is weakly but significantly associated with mean annual precipitation (a) but not associated with temperature (b) or the intensity of extreme wetness (c), here from SPEI03. Each point represents a site.

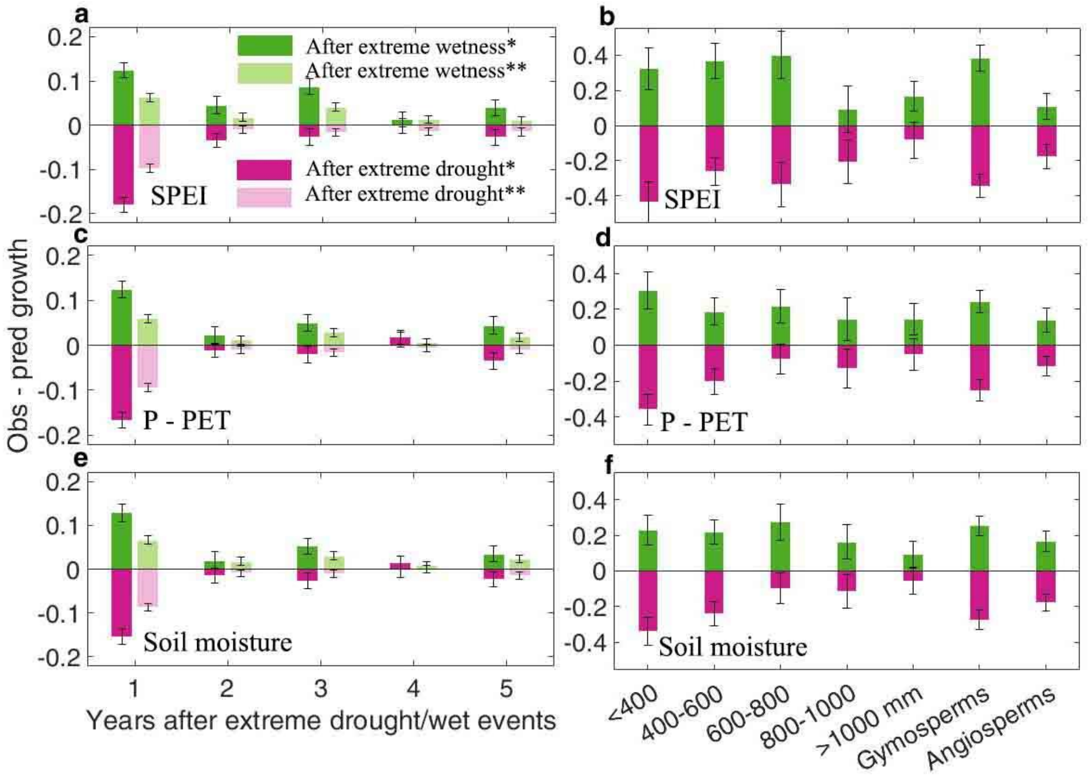

Supplementary Figure 8. Radial growth change after extreme wetness. Similar radial growth change patterns were also observed after using three different precipitation data sets (CRU, GPCC, PREC) to define extreme drought/wetness events. Error bars represent the 95% confidence intervals around the mean from bootstrapping (n = 5000 resamplings).

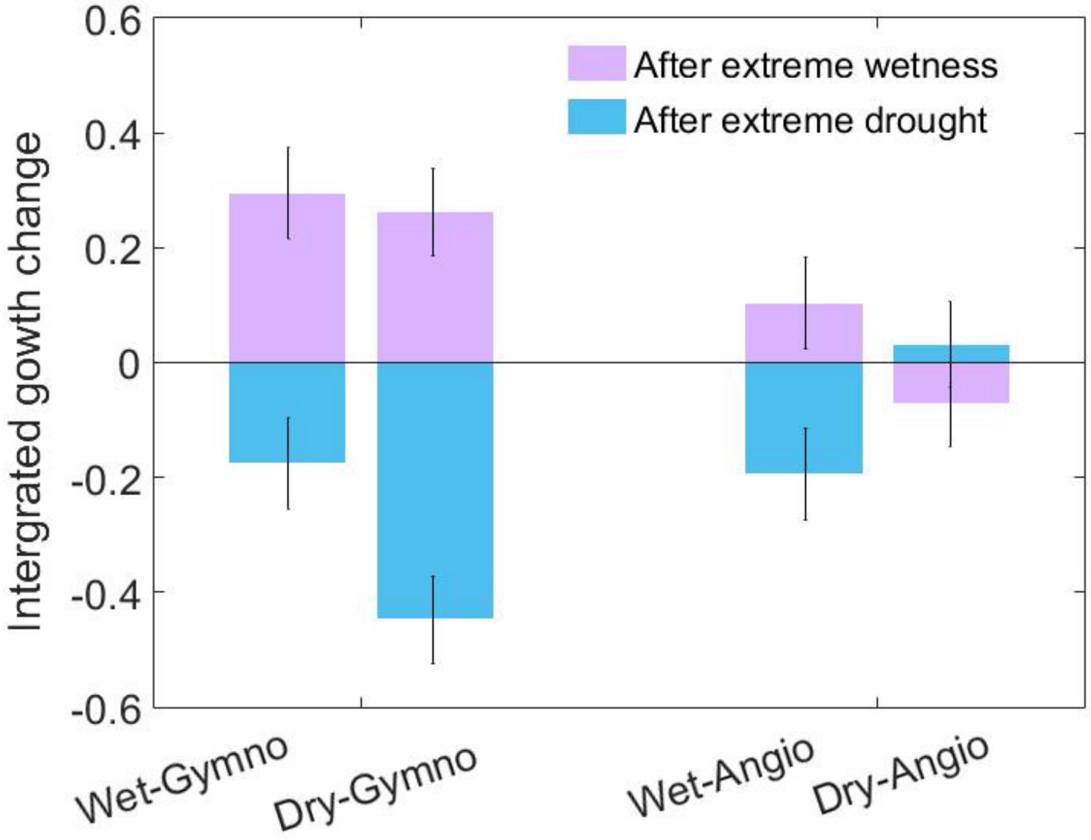

Supplementary Figure 9. Comparison of radial growth change between Gymnosperms and Angiosperms. Gymnosperms in both the dry (mean annual precipitation < 400 mm) and wet (mean annual precipitation > 800 mm) sites show substantial and positive enhanced growth after extreme wetness (exceeding the 95th quantile for SPEI03 value during the analysis period). By contrast, much lower enhanced growth is detected among the Angiosperms in the wet sites. Slight and reduced growths were detected among Angiosperms in dry sites. Error bars represent the 95% confidence intervals around the mean from bootstrapping (n = 5000 resamplings).

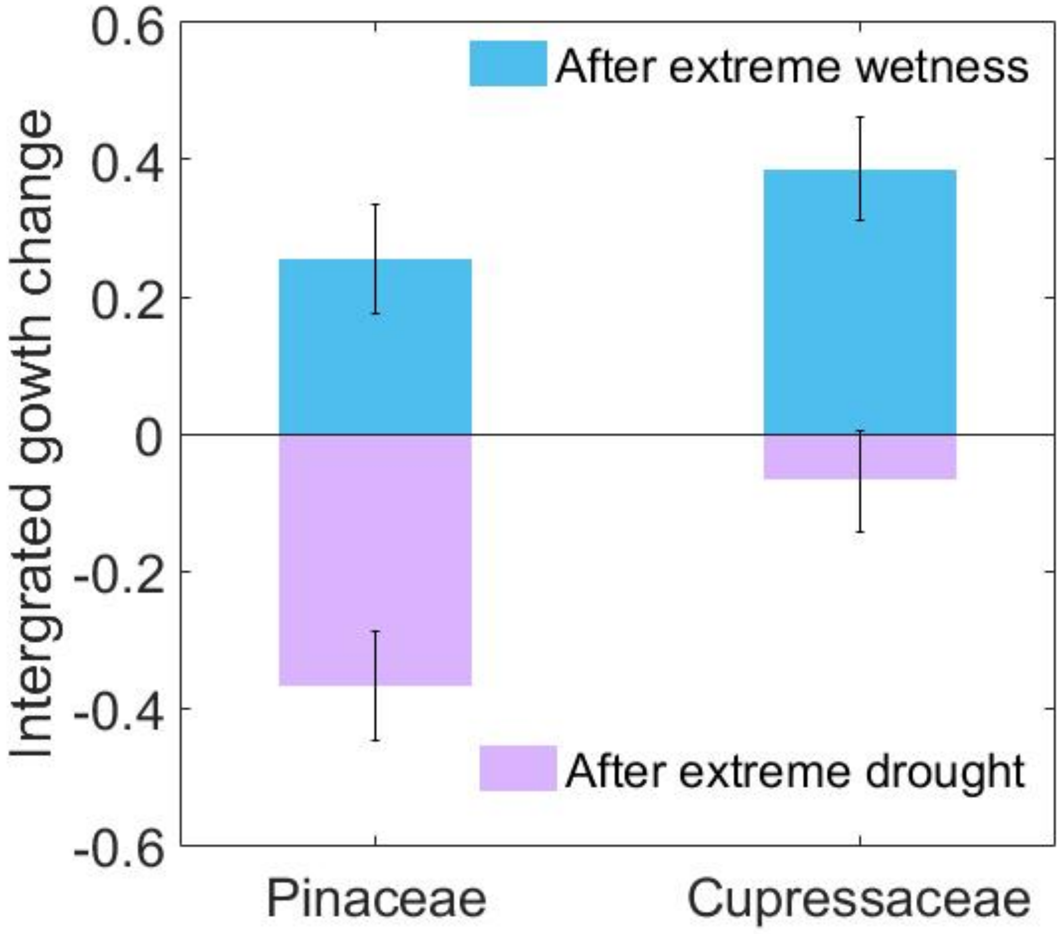

Supplementary Figure 10. Comparison of radial growth change between Pinaceae and Cupressaceae. Higher enhanced growth was detected in Cupressaceae than in Pinaceae using SPEI03. Error bars represent the 95% confidence intervals around the mean from bootstrapping (n = 5000 resamplings).

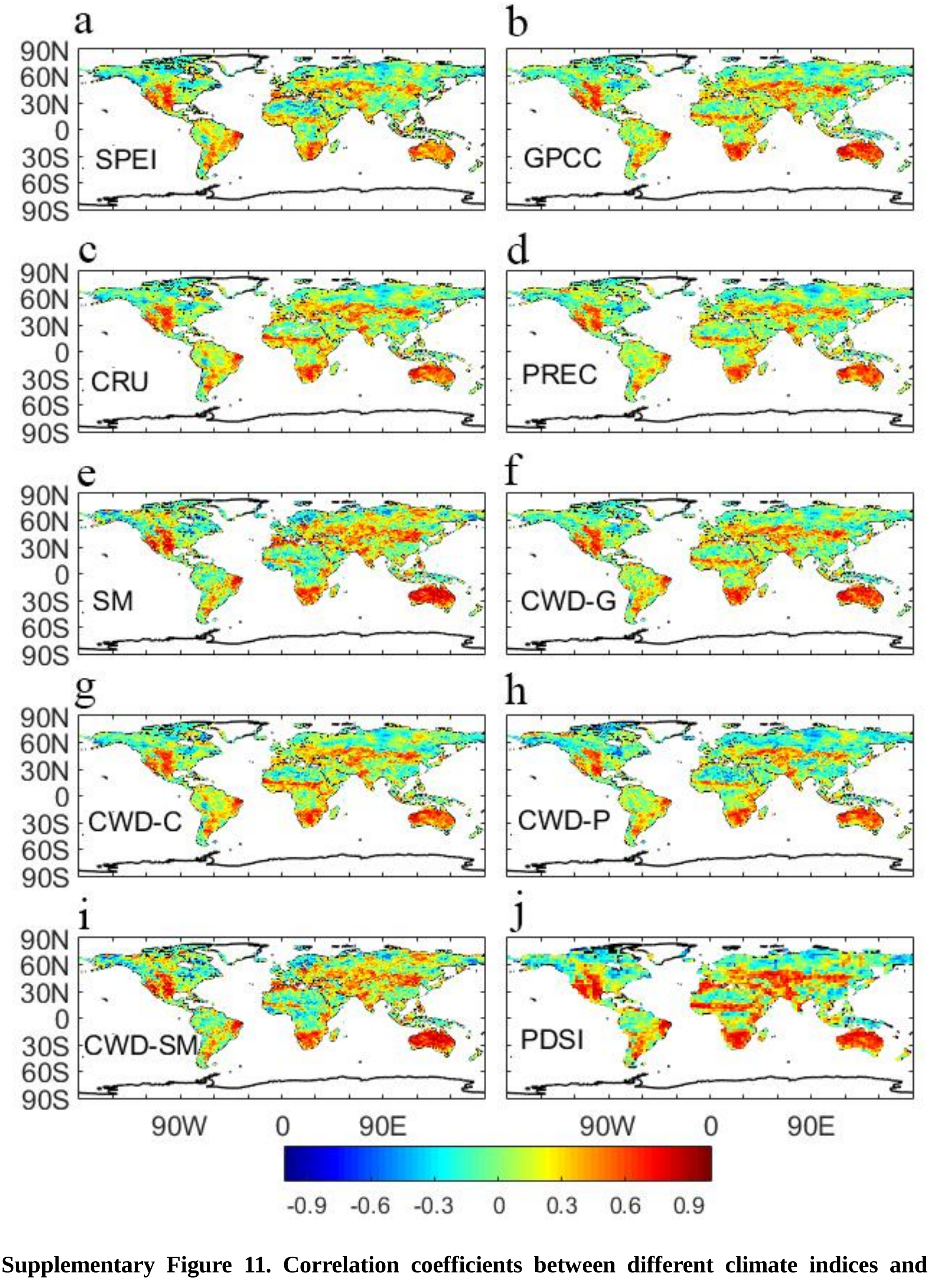

Supplementary Figure 11. Correlation coefficients between different climate indices and NDVI. Similar spatial patterns in correlation coefficients are observed between different climate indices and the NDVI. Climate indices, SPEI (a), precipitation from GPCC (b), CRU (c), PREC (d), soil moisture (e); CWD-G (f), CWD-C (g), CWD-P (h) and CWD-SM(i), are the differences between the CRU, GPCC, PREC precipitation and soil moisture (CPC) minus the potential evapotranspiration (PET), respectively; PDSI(j). Maps were created using Matlab R2015b

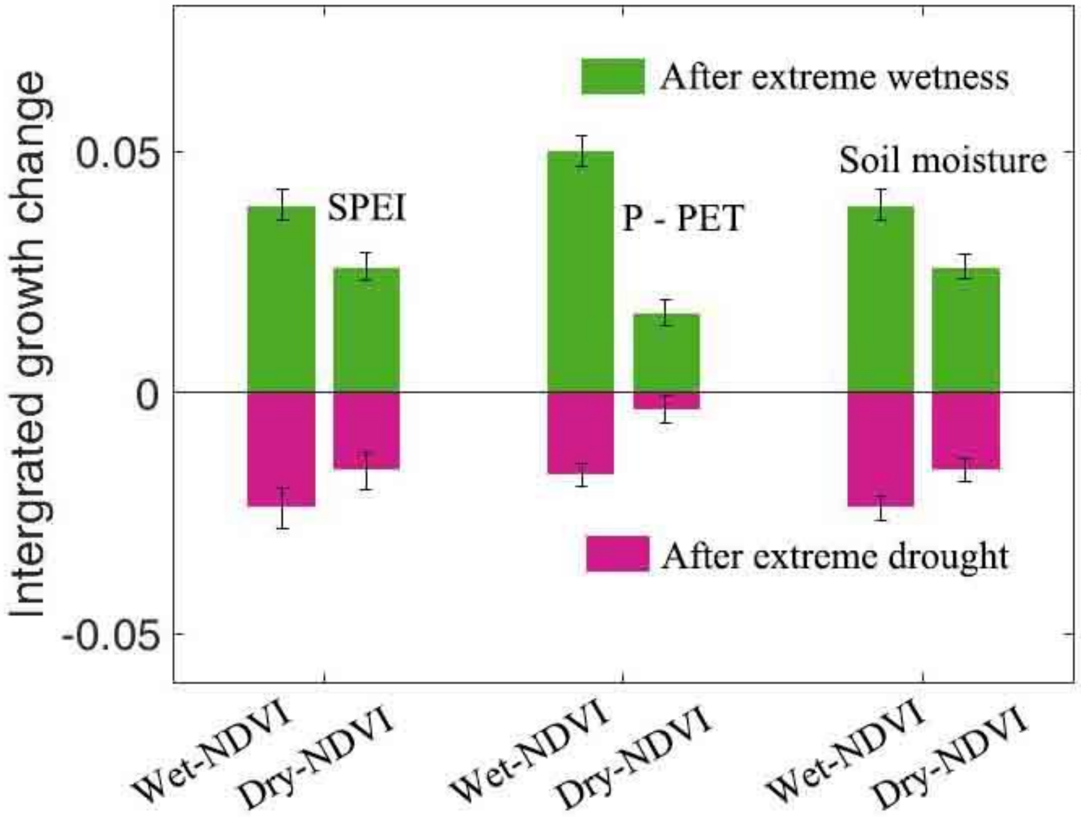

Supplementary Figure 12. NDVI change in the dry and wet regions. NDVI in dry regions shows higher enhanced greenness than that in wet regions. Error bars represent the 95% confidence intervals around the mean from bootstrapping (n = 5000 resamplings).

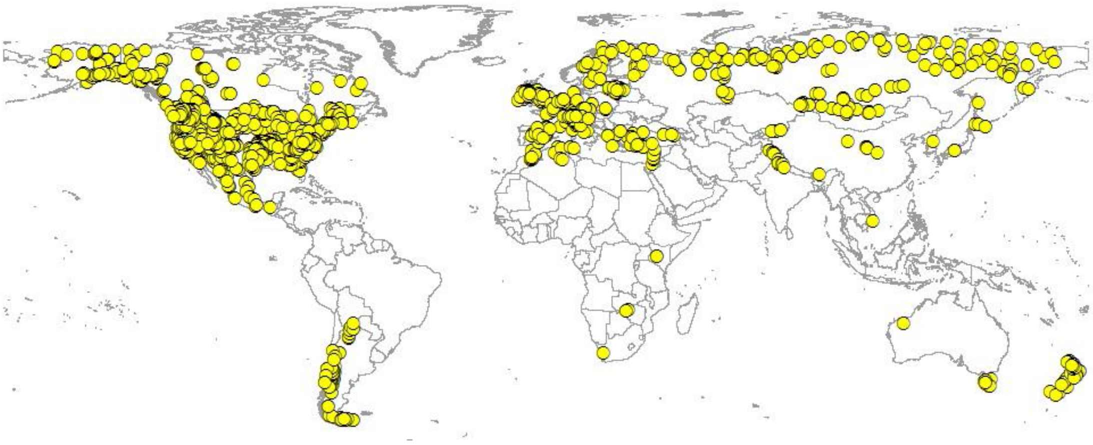

Supplementary Figure 13. Spatial distribution of the 1929 tree ring sites. Map was created using ArcMap 10.2

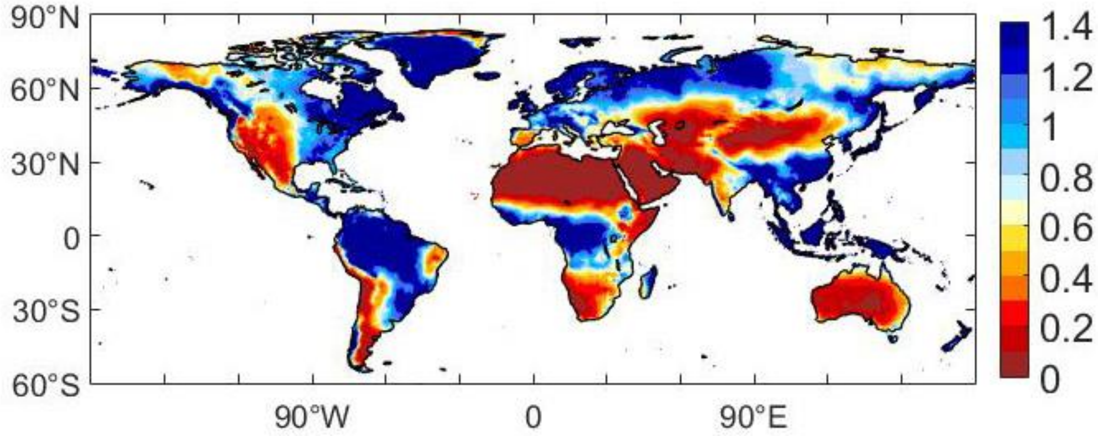

Supplementary Figure 14. Spatial pattern of the AI (P/PET) index. Regions with AI < 0.65 were defined as drylands (see Methods). Map was created using Matlab R2015b

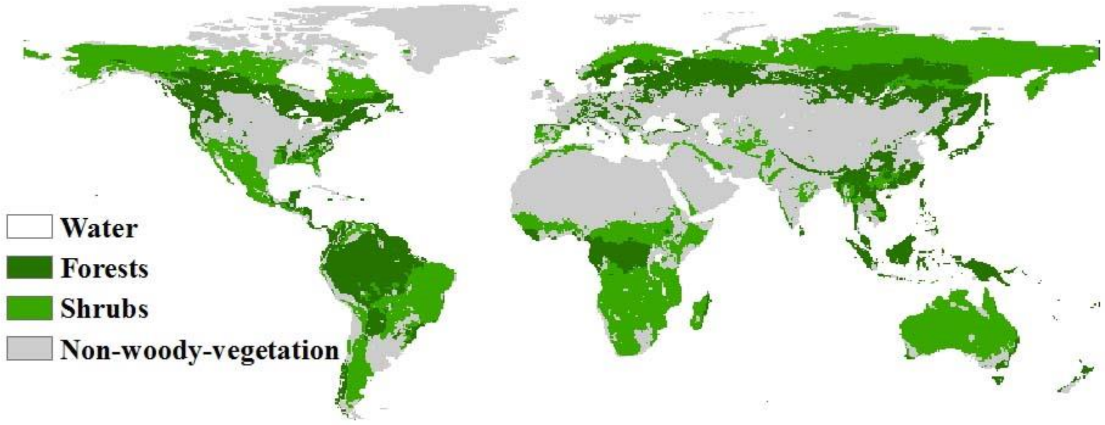

Supplementary Figure 15. Grouped land cover. Land cover was based on the International Geosphere-Biosphere Program (IGBP) scheme Moderate Resolution Imaging Spectroradiometer land cover product MOD12C1. We grouped evergreen broadleaf forest, evergreen needle-leaf forest, deciduous needle-leaf forest, deciduous broadleaf forest and mixed forest as forests and closed shrub lands, open shrub lands, woody savannas, and savannas as shrubs, and the other land cover types were grouped as non-woody-vegetation. Map was created using ArcMap 10.2

Supplementary Table 1. Observed change percentages versus predicted tree radial growth after extreme wetness and drought using three different climate indices based on a linear climate-growth model.

Climate indices	Enhanced growth (%)	Reduced growth (%)	Compensation ratio (%)
SPEI	22±7	-26±8	85
P – PET	21±6	-20±7	105
Soil moisture	10±5	-11±5	91
Average	18±6	-19±7	93

Supplementary Table 2. Observed change percentages versus predicted tree radial growth after extreme drought in arid and wet forests using three different climate indices based on a linear climate-growth model.

Climate indices	Arid area (%)	Wet area (%)
SPEI	-40±7	-7±6
P – PET	-35±6	-3±3
Soil moisture	-12±5	-1±4

Supplementary Table 3: Species and distributions that experienced extreme wetness and extreme drought during the time period studied (1948-2013) included in the ITRDB data sets.

Group	***Genus***	***Species***	***Regions***
Angiosperms	*Acer*	*saccharum*	*eastern North America*
Angiosperms	*Quercus*	*alba*	*eastern North America, Europe*
Angiosperms	*Quercus*	*douglasii*	*western North America*
Angiosperms	*Quercus*	*kelloggii*	*western North America*
Angiosperms	*Quercus*	*lobata*	*western North America*
Angiosperms	*Quercus*	*lyrata*	*eastern North America*
Angiosperms	*Quercus*	*macrocarpa*	*western North America, eastern North America*
Angiosperms	*Quercus*	*montana*	*eastern North America*
Angiosperms	*Quercus*	*mongolica*	*Asia*
Angiosperms	*Quercus*	*petraea*	*Europe*
Angiosperms	*Quercus*	*pubescens*	*Europe*
Angiosperms	*Quercus*	*stellata*	*eastern North America*
Angiosperms	*Quercus*	*robur*	*Europe*
Gymnosperms	*Abies*	*alba*	*Europe*
Gymnosperms	*Abies*	*cilicica*	*Asia*
Gymnosperms	*Abies*	*lasiocarpa*	*western North America*
Gymnosperms	*Abies*	*procera*	*western North America*
Gymnosperms	*Abies*	*pindrow*	*Asia*
Gymnosperms	*Juniperus*	*occidentalis*	*western North America, eastern North America*
Gymnosperms	*Juniperus*	*phoenicea*	*Asia*
Gymnosperms	*Juniperus*	*virginiana*	*eastern North America*
Gymnosperms	*Larix*	*kurulensis*	*eastern Siberia*
Gymnosperms	*Larix*	*sibirica*	*eastern Siberia*
Gymnosperms	*Larix*	*lyallii*	*western North America*

Gymnosperms	*Larix*	*decidua*	*Europe*
Gymnosperms	*Phyllocladus*	*aspleniifolius*	*Australia*
Gymnosperms	*Picea*	*engelmann*	*western North America*
Gymnosperms	*Picea*	*glauca*	*western Canada, western North America*
Gymnosperms	*Picea*	*orientalis*	*Asia*
Gymnosperms	*Picea*	*rubens*	*eastern North America*
Gymnosperms	*Picea*	*sitchensis*	*Alaska*
Gymnosperms	*Pinus*	*albicaulis*	*western North America*
Gymnosperms	*Pinus*	*balfouriana*	*western North America*
Gymnosperms	*Pinus*	*banksiana*	*western North America*
Gymnosperms	*Pinus*	*cembroides*	*western North America*
Gymnosperms	*Pinus*	*cembra*	*Europe*
Gymnosperms	*Pinus*	*contorta*	*western North America*
Gymnosperms	*Pinus*	*edulis*	*western North America*
Gymnosperms	*Pinus*	*echinata*	*eastern North America*
Gymnosperms	*Pinus*	*flexilis*	*western North America*
Gymnosperms	*Pinus*	*ponderosa*	*western North America*
Gymnosperms	*Pinus*	*sibirica*	*Siberia*
Gymnosperms	*Pinus*	*strobus*	*eastern North America*
Gymnosperms	*Pseudotsuga*	*macrocarpa*	*western North America*
Gymnosperms	*Pseudotsuga*	*menziesii*	*western North America*
Gymnosperms	*Taxodium*	*distichum*	*eastern North America*
Gymnosperms	*Tsuga*	*canadensis*	*eastern North America*
